# Supplementary figures and images for: Trynity controls epidermal barrier function and respiratory tube maturation in Drosophila by modulating apical extracellular matrix nano-patterning
Source: PLoS One. 2018 Dec 21;13(12):e0209058. doi: 10.1371/journal.pone.0209058 (PMC6303098; doi:10.1371/journal.pone.0209058)

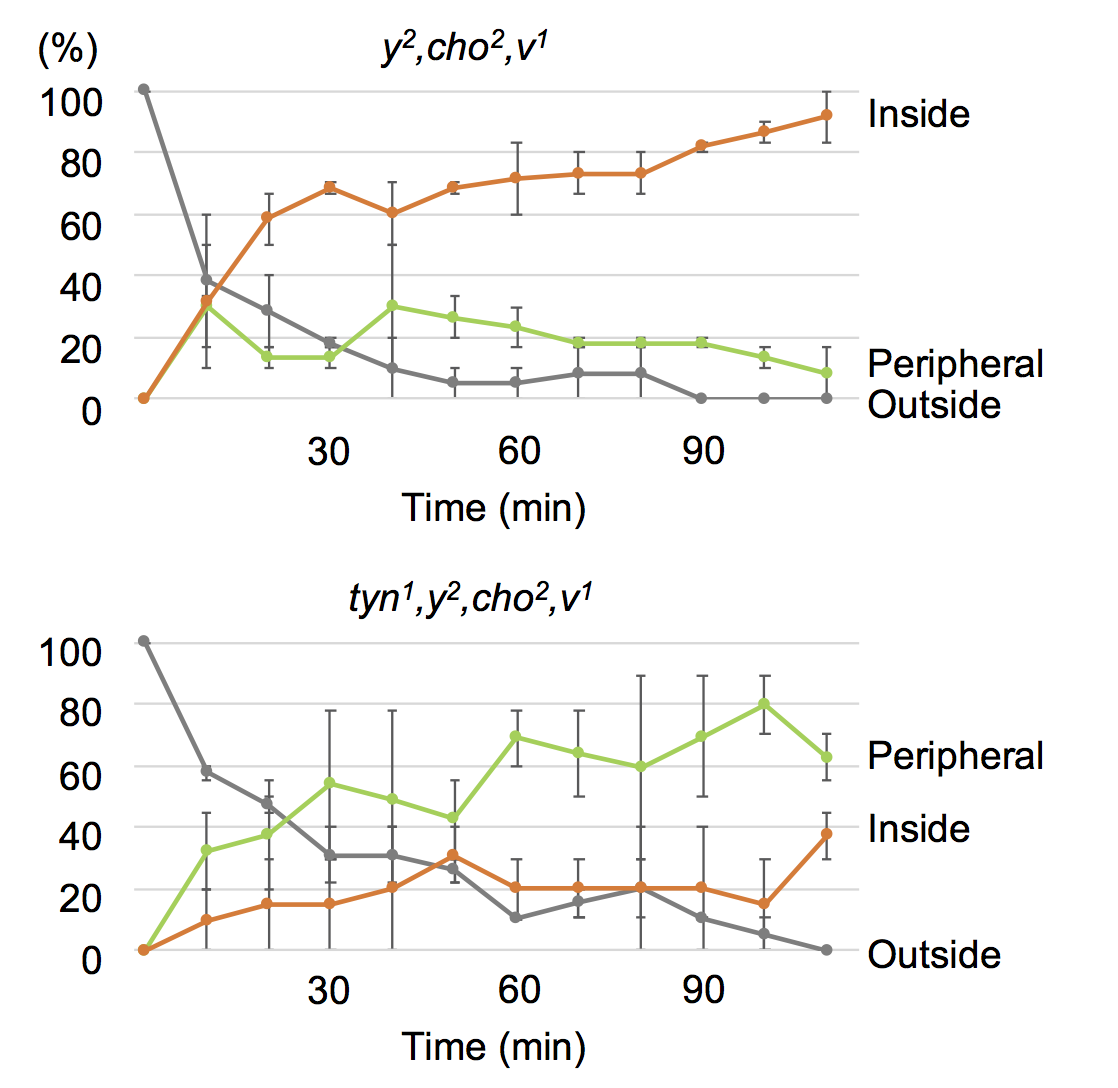

Supplement: S1 Fig — Yeast paste was placed at the center of the well at time 0. (Upper) The numbers of y2, cho2, v1 larvae inside and outside the paste increased and decreased, respectively, meaning that they gradually moved into the yeast paste. Approximately 20% of the larvae were peripheral to the yeast paste at any time point. (Lower) tyn1 larvae outside the paste decreased similarly to y2, cho2, v1, indicating that the tyn1 larvae could sense and move toward the food. However, more larvae tended to stay at the periphery of the food rather than entering it, compared to the control. (TIFF) [file pone.0209058.s001.tiff]

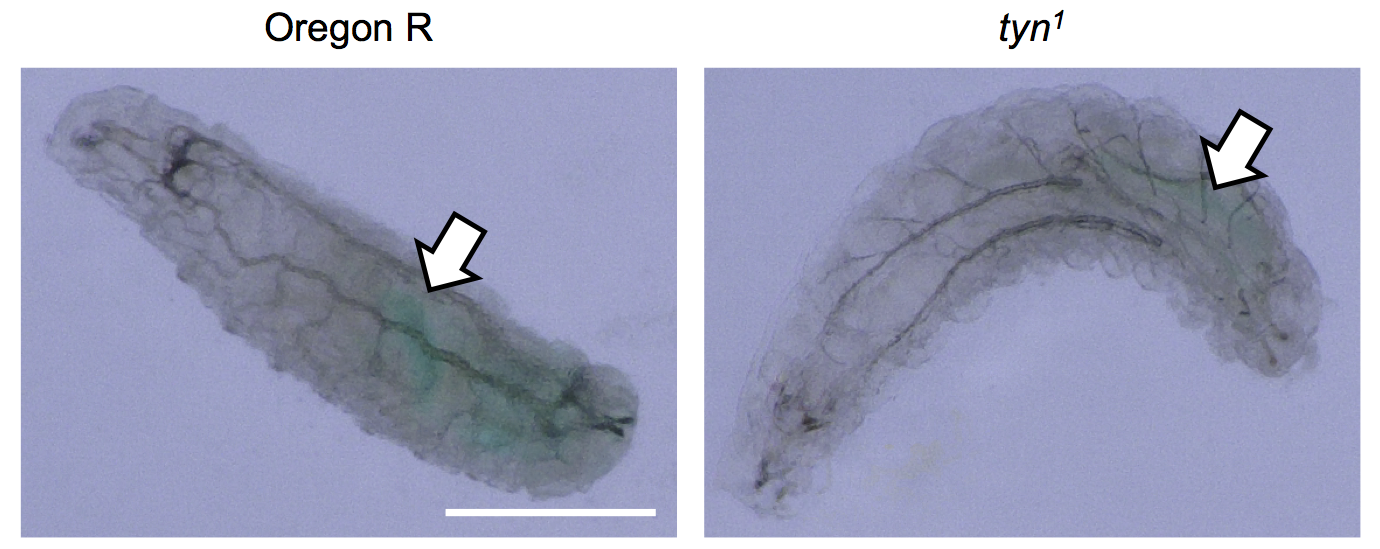

Supplement: S2 Fig — Images of Oregon R (left) and tyn1 (right) newly-hatched larvae 30 min. after applying yeast paste with 0.5% Brilliant Blue FCF. Arrows indicate the blue-colored guts, showing the abilities for food intake. The tyn1 larva shows the partial gas-filling defect. Scale bar: 200 μm. (TIFF) [file pone.0209058.s002.tiff]

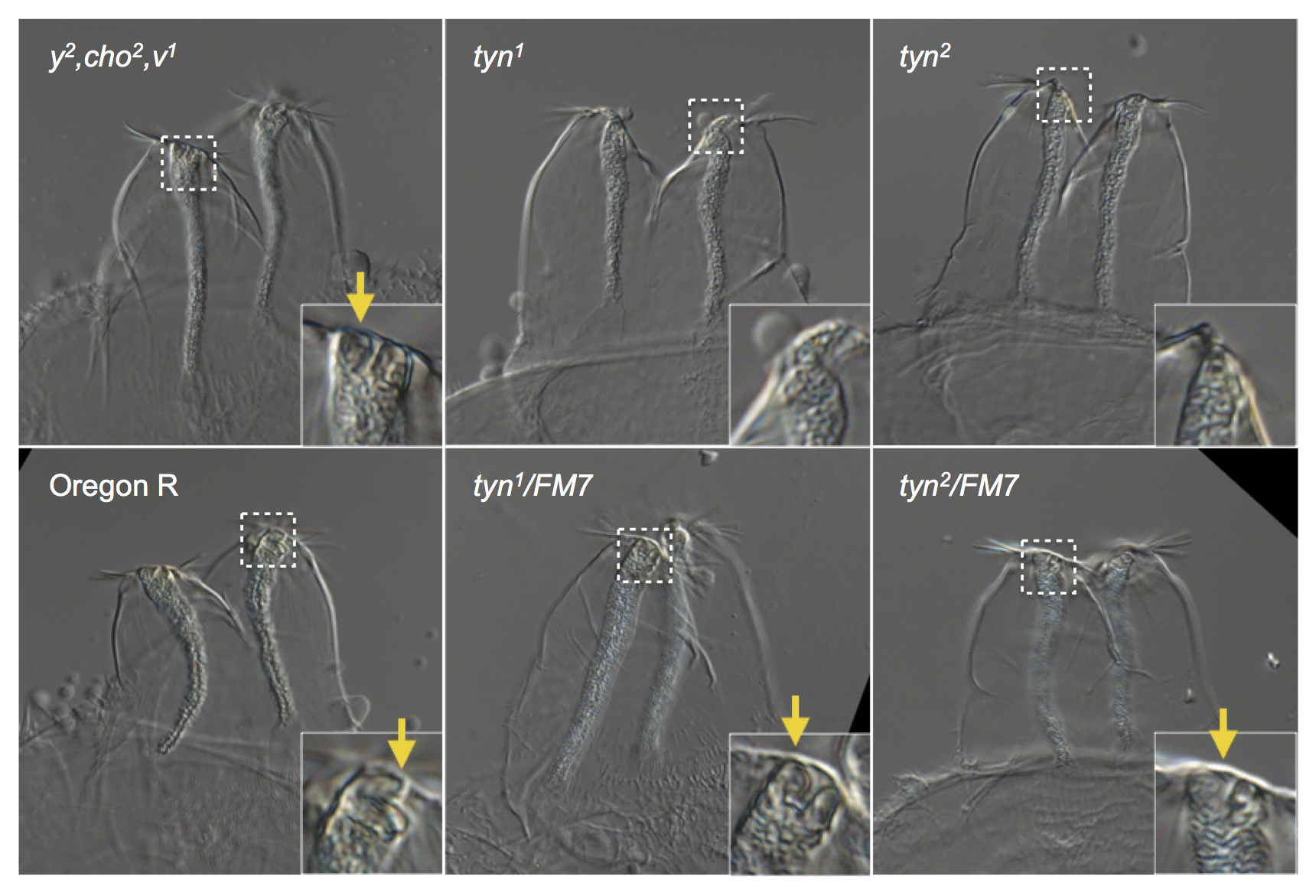

Supplement: S3 Fig — Posterior spiracles with enlarged views of the posterior tip (dotted-lined region) in the lower right corner are shown for various genotypes (y2, cho2, v1, Oregon R, tyn1, tyn2 and their sibling controls). The yellow arrows indicate valve structures, which were missing in the tyn1 and tyn2 mutants. (TIFF) [file pone.0209058.s003.tiff]

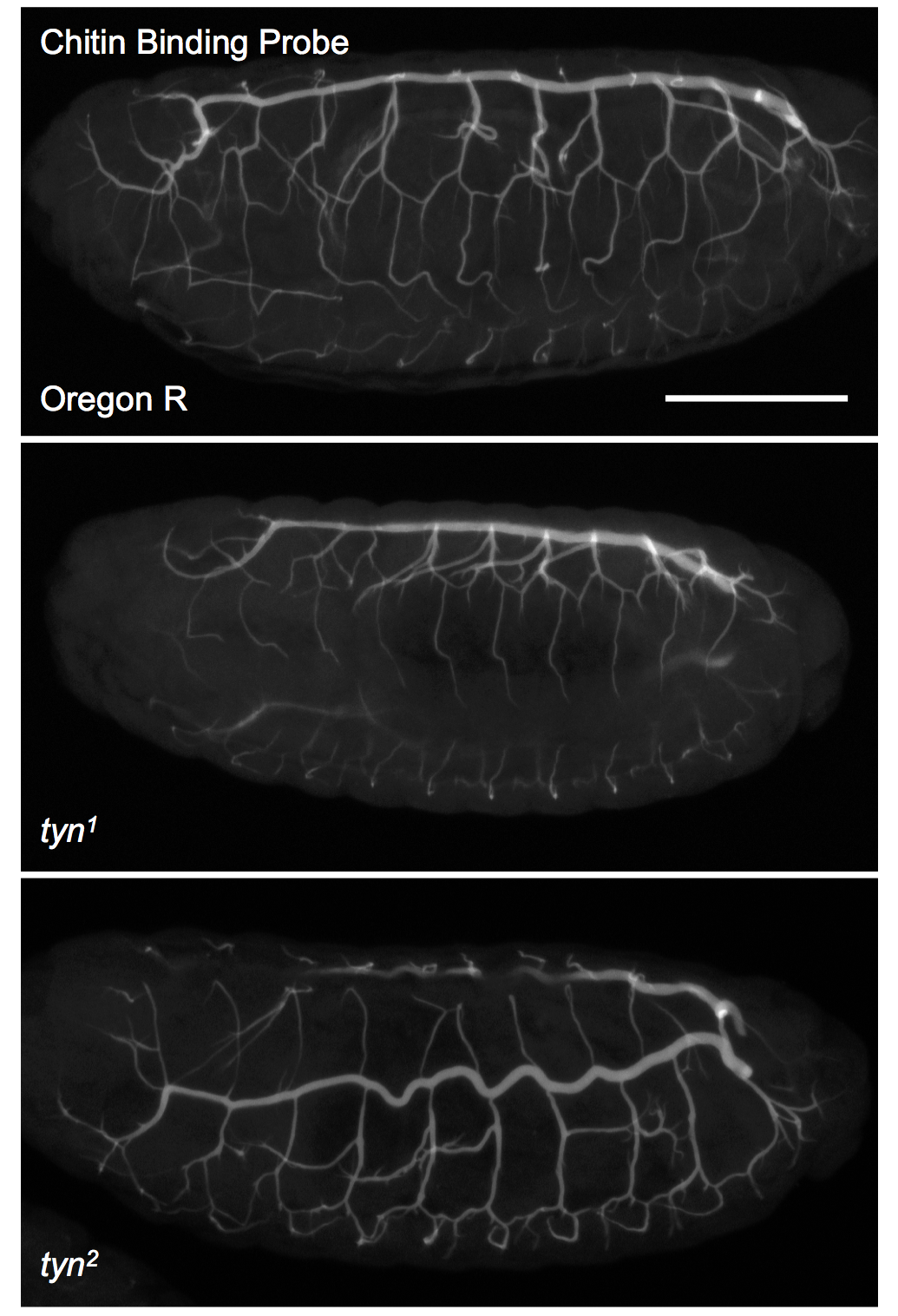

Supplement: S4 Fig — Oregon R, tyn1, and tyn2 embryos were labeled with chitin-binding probe. All of the three genotypes showed similar Chitin pattern in tracheal system. Lateral views, anterior to the left and dorsal to the top. Scale bar: 100 μm. (TIFF) [file pone.0209058.s004.tiff]
